# Supplementary material for: Oncolytic Tanapoxvirus Variants Expressing mIL-2 and mCCL-2 Regress Human Pancreatic Cancer Xenografts in Nude Mice
Source: Biomedicines. 2024 Aug 12;12(8):1834. doi: 10.3390/biomedicines12081834 (PMC11351728; doi:10.3390/biomedicines12081834)
Supplement: Supplementary file 1 [file biomedicines-12-01834-s001.zip › biomedicines-3003347-supplementary.pdf]

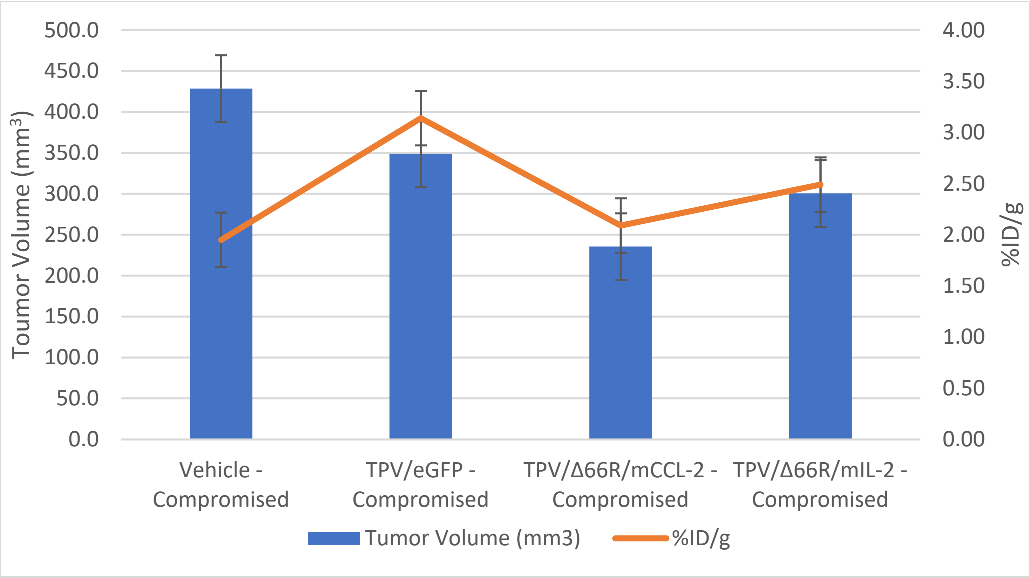

A

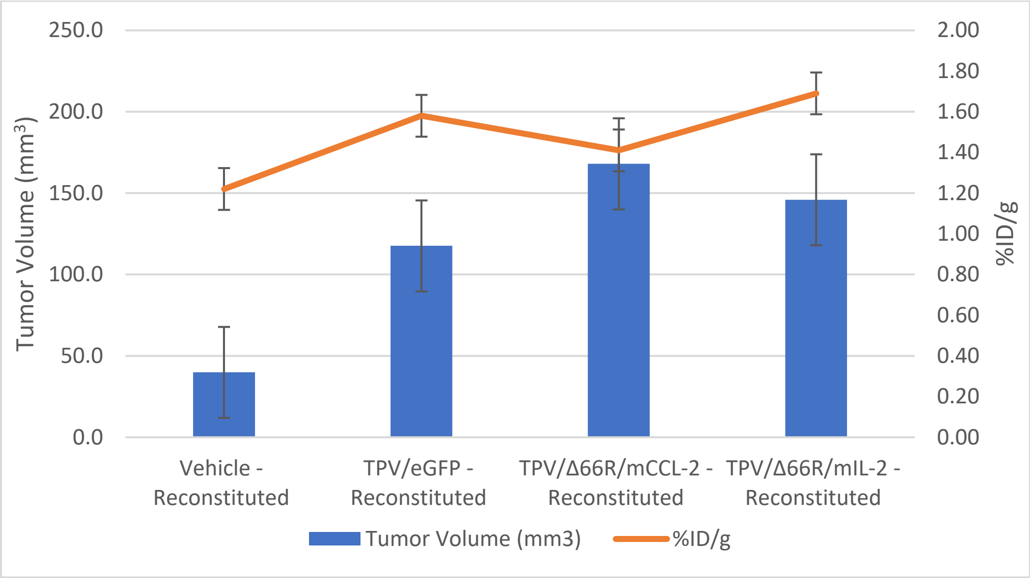

B

Figure S1. Immunocompromised and Immune Reconstituted Group Mean Tumor Volume and [^{125}I]-anti-eGFP or [^{125}I]-anti-mCherry Antibody SPECT/CT Percent Injected Dose per Gram Tissue

BxPc-3 human PDAC cells were inoculated subcutaneously (SC) in the right flank region of immunocompetent, female, BALB-c Nude Mice. Each subject received a single inoculation of 5 x 10^6 cells / subject. Each subject was treated with a single, intratumoral administration of 100 µL PBS formulation vehicle or 100X virus stock of each applicable recombinant virus to result in a virus mass dose of 5 x 10^6 plaque forming units (pfu) per animal when tumors reached approximately 200 mm^3. SPECT/CT images were acquired during Week 7 – 8, 48-hours post imaging agent administration (200 µCi/subject). Bars show standard error of the mean (±1 SEM). Immunocompromised (A) and immune reconstituted (B), intergroup statistical comparisons resulted

in lack of statistical significance for all comparisons and do not demonstrate tumor-volume dependency.

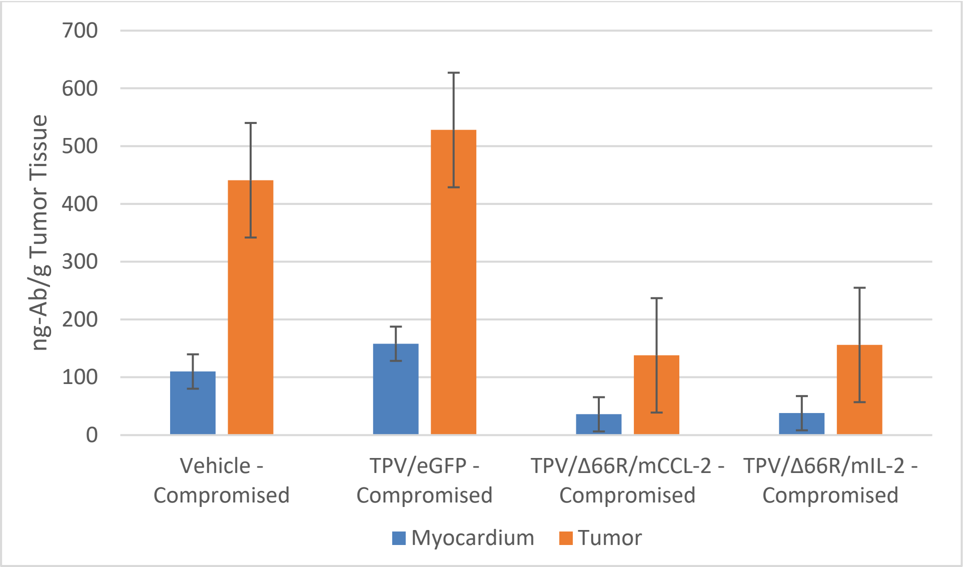

A

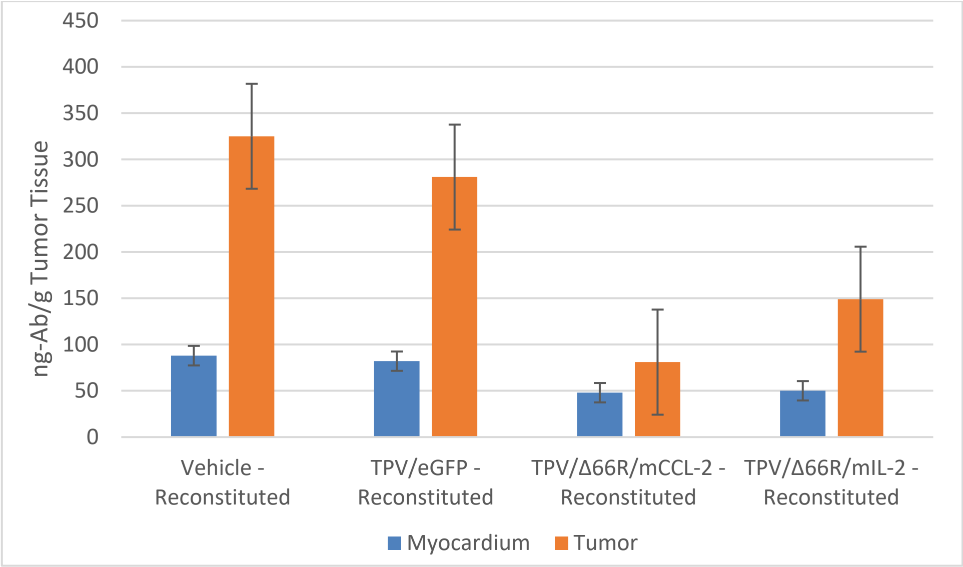

B

Figure S2. Immunocompromised and Immune Reconstituted [^{125}I]-anti-eGFP or [^{125}I]-anti-mCherry Antibody Quantitative Whole Body Autoradiography Nanogram-antibody per Gram Tissue

BxPc-3 human PDAC cells were inoculated subcutaneously (SC) in the right flank region of immunocompetent, female, BALB-c Nude Mice. Each subject received a single inoculation of 5 x 10^6 cells / subject. Each subject was treated with a single, intratumoral administration of 100 µL PBS formulation vehicle or 100X virus stock of each applicable recombinant virus to result in a virus mass dose of 5 x 10^6 plaque forming units (pfu) per animal when tumors reached

approximately 200 mm^3. QWBA images were acquired 48-hours post imaging agent administration (200 µCi/subject). Bars show standard error of the mean (±1 SEM). Immunocompromised (A) and immune reconstituted (B), intergroup statistical comparisons resulted in lack of statistical significance between tumor and control tissue within and between study groups.

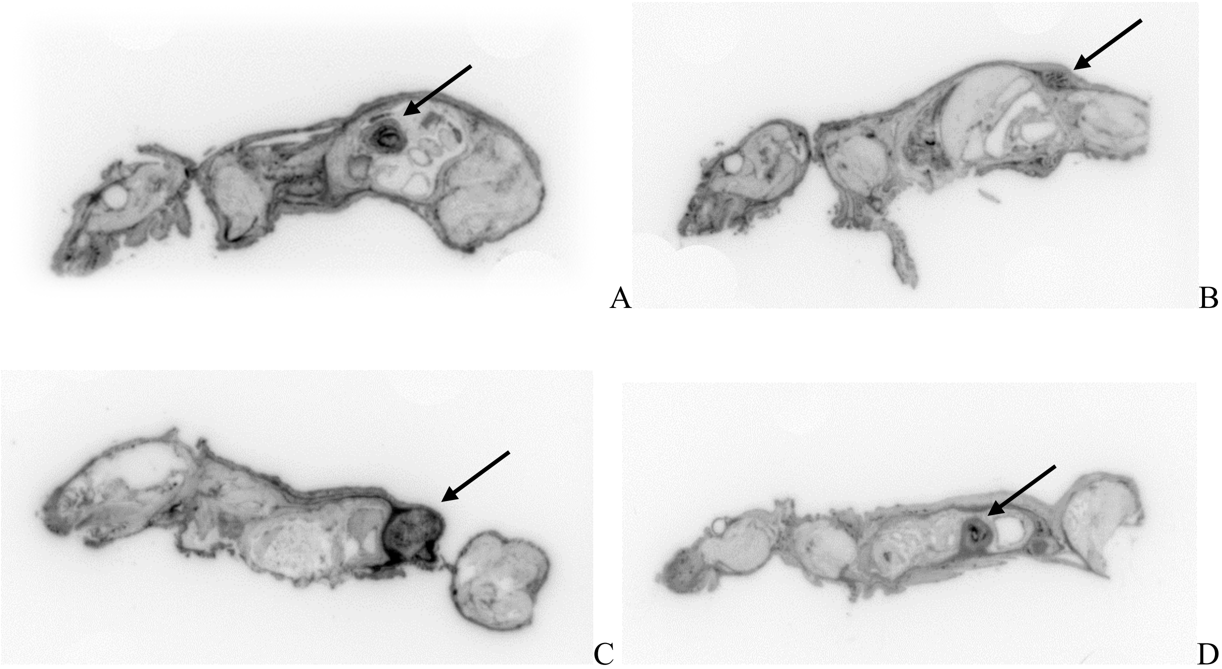

Figure S3. [^{125}I]-anti-eGFP or [^{125}I]-anti-mCherry Antibody Quantitative Whole Body Autoradiography Images of BxPc-3 PDAC Human Tumor Xenografts in Immunocompromised and Immune Reconstituted BALB-c Nude Mice
BxPc-3 human PDAC cells (5 x 10^6 cells / subject) were inoculated subcutaneously (SC) in the right flank region of immunocompromised or immune reconstituted, female, BALB-c Nude Mice. Each subject was treated with a single, intratumoral administration of 100 µL PBS formulation vehicle or 100X virus stock of each applicable recombinant virus to result in a virus mass dose of 5 x 10^6 plaque forming units (pfu) per animal when tumors reached approximately 200 mm^3. Autoradioluminograms were generated from subjects processed for whole-body autoradiography immediately following completion of SEPCT/CT during Weeks 7 – 8, at 48-hours post imaging agent administration (200 µCi/subject). Select, representative QWBA images as follows: immunocompromised vehicle control (A), immune reconstituted vehicle control (B), TPV/eGFP immunocompromised (C) and TPV/eGFP immune reconstituted; black arrows indicate tumor location.

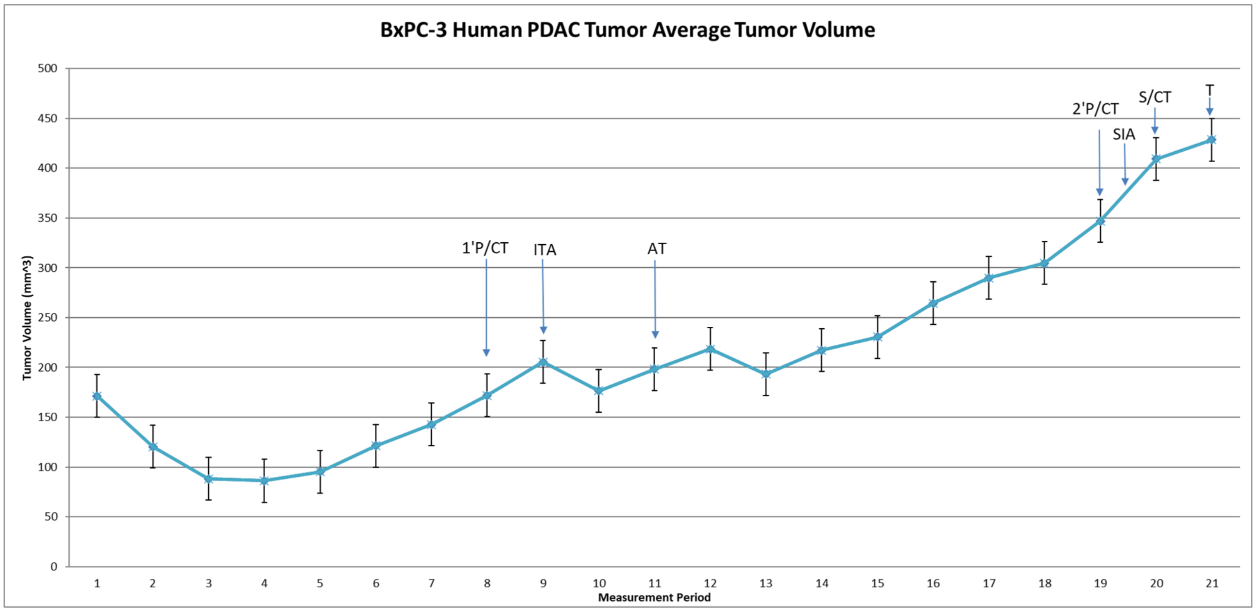

Figure S4. Key Study Events

Human pancreatic cancer cell (BxPc-3) growth curve in immunocompromised BALB-c (CAnN.Cg-F*oxn1*^{nu}*/Crl*) nude mice treated with phosphate buffered saline (PBS), 100 µL, via intratumoral administration. Graph demonstrates temporal relationship of key study activities through the in-life phase of each applicable study cohort; for those study cohorts receiving virotherapy, each applicable TPV recombinant was administered at the time of the vehicle treatment displayed here for the control group. Measurements were performed every three days post tumor cell inoculation. Key: 1'P/CT = primary PET/CT acquisition; ITA = intratumoral treatment administration (vehicle or TPV recombinant); AT = adoptive T-cell transfer when applicable; 2'P/CT = secondary PET/CT acquisition; SIA = SPECT imaging agent administration; S/CT = SPECT/CT acquisition; T = termination of in-life phase.

Table S1. Study Design Summary

Group	Animal Number	Immune System Status	Treatment Condition	BxPc-3 Mass Dose / Animal	PET/CT Imaging Agent	SPECT/CT Imaging Agent	Primary Endpoints
1	18	D	NA	5×10^6 cells	NA		Tumor Volume
2	9	D	Vehicle		[^{18}F]-FDG & [^{18}F]-FLT	[^{125}I] α-GFP	Tumor Volume, PET, SPECT, QWBA and Histology
3	9	C	Vehicle		[^{18}F]-FDG & [^{18}F]-FLT	[^{125}I] α-GFP	
4	9	D	TPV/eGFP		[^{18}F]-FDG & [^{18}F]-FLT	[^{125}I] α-GFP	
5	9	C	TPV/eGFP		[^{18}F]-FDG & [^{18}F]-FLT	[^{125}I] α-GFP	
6	9	D	TPV/Δ66R/mCCL-2		[^{18}F]-FDG & [^{18}F]-FLT	[^{125}I] α-mCherry	
7	9	C	TPV/Δ66R/mCCL-2		[^{18}F]-FDG & [^{18}F]-FLT	[^{125}I] α-mCherry	
8	9	D	TPV/Δ66R/mIL-2		[^{18}F]-FDG & [^{18}F]-FLT	[^{125}I] α-mCherry	
9	9	C	TPV/Δ66R/mIL-2		[^{18}F]-FDG & [^{18}F]-FLT	[^{125}I] α-mCherry	

NOTE: Group 1 animals received BxPc-3 cell inoculation only; these subjects were assessed via in-life observations and direct tumor measurements for a minimum of 28 days following inoculation to establish cell/tumor growth rate. Animals in Groups 2 – 9 were split into two cohorts of n = 4 – 5 subjects to be imaged via PET Imaging with either [^{18}F] FDG or [^{18}F] FLT prior to and following virotherapy at approximately 21- and 60-days respectively. SPECT/CT imaging was conducted on n=3 study subjects assigned to Groups 2-9 at approximately 63-days post virotherapy. QWBA imaging was conducted on the cohort of n=3 study subjects from Groups 2-9 selected for SPECT/CT imaging following completion SPECT/CT imaging. Key: μCi – microcurie; D- immunodeficient; C- immune reconstituted; PET – Positron Emission Tomography; CT – Computed Tomography; SPECT – Single Photon Emission Tomography; [^{125}I] α – iodine 125 radiolabeled antibody targeting either eGFP or mCherry; [^{18}F]-FDG - [^{18}F]-fluorodeoxyglucose; and [^{18}F]-FLT - [^{18}F]-fluoro-3’-deoxy-3’-L:flouorothymidine.
